# The Impact of Children’s and Parents’ Perceptions of Parenting Styles on Attention, Hyperactivity, Anxiety, and Emotional Regulation

**DOI:** 10.3390/children11030313

**Published:** 2024-03-06

**Authors:** Marisol Cueli, Natalia Martín, Laura M. Cañamero, Celestino Rodríguez, Paloma González-Castro

**Affiliations:** Department of Psychology, University of Oviedo, Plaza Feijoo S/N, 33003 Oviedo, Spain; cuelimarisol@uniovi.es (M.C.); uo17835@uniovi.es (N.M.); canamerol@uniovi.es (L.M.C.); mgcastro@uniovi.es (P.G.-C.)

**Keywords:** ADHD, parenting style, warmth–communication, criticism–rejection, attention, hyperactivity, anxiety, emotional regulation

## Abstract

Attention-deficit/hyperactivity disorder (ADHD) symptomatology can be studied by examining the associated neurobiological factors or by looking at the environmental factors involved, such as parenting styles. Negative parenting styles have been associated with ADHD symptoms in childhood and adolescence. The present study aimed to analyze the predictive power of two parenting style dimensions (warmth–communication and criticism–rejection) and three factors about rule-setting and compliance (inductive, strict, and indulgent styles) in the explanation of ADHD symptoms (attention and hyperactivity) and associated emotional factors (anxiety and emotional regulation) considering parents’ and children’s perspectives. The results indicate that from the parents’ perspective, the criticism–rejection variable was the most important in explaining attention difficulties, anxiety and emotional regulation. From the children’s perspective, the strict parenting style was the most important variable in explaining hyperactivity and emotional regulation. In addition, for children, warmth–communication was significant in predicting fewer emotional regulation difficulties. Our results highlight the importance of considering family dynamics when assessing ADHD in order to implement comprehensive interventions that consider parental training in positive parenting styles.

## 1. Introduction

Attention-deficit/hyperactivity disorder (ADHD) is a neurodevelopmental disorder characterized by a persistent pattern of inattention, hyperactivity, and impulsivity that is very common in children and adolescents, with studies reporting a prevalence ranging between 5.9 and 7.1% in this population [1,2]. In addition to the three main deficits, inattention, hyperactivity, and impulsivity, difficulties in working memory and response inhibition have also often been described in individuals with ADHD [3]. Looking beyond deficits in particular skills, over recent years, research into executive dysfunction has played a prominent role in discussions about the core deficit in ADHD [3,4]. There have been attempts to explain the basis of ADHD symptoms considering the neurobiological factors involved (neuroanatomical and neurophysiological). For example, at the neuroanatomical level, neuroimaging studies have reported reduced activation in certain brain regions (such as the dorsolateral prefrontal cortex, anterior cingulate cortex, posterior parietal cortex, ventrolateral prefrontal cortex, insula thalamus, and striatum) in individuals with ADHD [5]. Other studies have reported abnormalities in cortical thickness in the lateral prefrontal and parietal cortices in adolescents with ADHD, as well as alterations in the gray matter volume of the thalamus [6,7]. At the neurophysiological level, altered theta and beta ratios in frontal areas have been observed in electroencephalography in children and adolescents with ADHD [8].

In addition to studying the basis of the disorder, another line of research has focused on the factors that affect ADHD severity, including not only neurobiological factors but also children’s developmental contexts. The fifth edition of the Diagnostic and Statistical Manual of Mental Disorders added a specification of the severity of ADHD to its previous entry, differentiating between mild, moderate, and severe levels [9].

Arildskov et al. [10] analyzed the relationship between the severity of ADHD symptoms and impairment and found a gradual linear increase in impairment with greater symptom severity. The severity of the disorder may be affected by the presence of comorbid symptomatology [10,11] and by concurrent emotional symptomatology. Sasser et al. [12] examined the progression of ADHD symptomatology and found that it did not persist during adolescence in every case, but instead there was greater severity and persistence of symptoms in those who had more hyperactive or aggressive behaviors or whose parents used inconsistent parenting practices with little discipline. Other studies have associated ADHD symptomatology with other parenting practices based on overprotection, control, parental rejection, high levels of discipline, and low levels of parental involvement [13,14,15,16]. Bearing this in mind, it is worth asking to what extent parenting practices may be associated with children’s symptomatology not only at the attentional level or in terms of hyperactivity but also at the emotional level.

Parenting styles refer to the patterns of care, upbringing, control and establishment of rules, warmth, and punishment that parents most often use in various contexts over time to educate their children, relate to them, and manage their behavior. According to Baumrind [17], parenting styles may be classified by considering two fundamental dimensions: affect (parents’ abilities to respond to, support, and pay attention to their children’s needs) and control (supervision, seeking to produce and maintain suitable behavior from the children). With those dimensions in mind, Baumrind differentiated between three parenting styles: authoritarian, democratic, and permissive [17]. These three parenting styles use different strategies to establish discipline. The authoritarian style is based on coercive discipline, with rigid formulas for relationships with the children, placing great importance on obedience and the use of punishment. The democratic style uses inductive discipline that encourages rational, interactive strategies using reasoning to explain rules and requirements. The permissive style is based on indulgent discipline that prompts the child to participate in shaping rules, without using punishment, and presenting a high level of tolerance. 

Based on the interpersonal acceptance–rejection theory [18], which is in line with Baumrind’s view, parental warmth and behavioral control are two primary forms of parenting with long-term implications for children’s development. Each of these parenting styles has a different impact on the child’s psychosocial development and adjustment, and this is even more important in children with ADHD. Longitudinal studies such as Brinksma et al.’s [14] have emphasized the factors that are associated with the severity or persistence of ADHD symptomatology and have linked the higher severity and longer persistence of ADHD with parenting styles based on rejection. Brinksma et al. [14] concluded that children’s perceptions of parental rejection were a clear predictor of the persistence of ADHD symptomatology. In addition, Kim et al. [16] linked negative parenting styles that used parental rejection, as perceived by the children, to more severe, more persistent symptoms of ADHD. In contrast, parental warmth has positive implications for the personal development and psychological adjustment of children with ADHD [14,19,20]. The parenting style can also have an impact on children’s levels of anxiety and their regulation of emotions. Some studies have shown that the parent–child relationship has a direct interaction with children’s anxiety [21,22]. Rothenberg et al. [23] reported that when parents showed coldness/lack of affection, hostility/aggression, indifference/neglect, and undifferentiated rejection, children reported higher externalizing and internalizing problems across most ages between 7 and 14.

In short, some studies have linked parenting styles to the amount and intensity of ADHD symptomatology, as well as to comorbidities and other functional difficulties associated with the disorder [13,14,16,24]. As far as we are aware, these studies have assessed parenting styles by considering the information provided by children [14,16] or by parents [20]. The objective of the present study was to examine the extent to which variables associated with parenting styles as perceived by children and by parents affected the presence of ADHD symptomatology and other emotional factors. More specifically, the study aimed to establish whether the parenting style (warmth–communication, criticism–rejection) and the strategies to establish discipline (inductive, rigid, indulgent) are more significant and which variables they affect, differentiating between symptoms associated with attention, hyperactivity, anxiety, and emotional regulation. The research question that the study aimed to address is whether some aspects of parenting styles—such as warmth–communication and criticism–rejection or indulgent strategies for establishing discipline—can predict difficulties in attention, hyperactivity, anxiety, or emotional regulation in children with ADHD.

## 2. Materials and Methods

### 2.1. Participants

The families of children with ADHD were invited to participate in the study. The criterium for participation was a prior diagnosis of ADHD. Participants were selected via ADHD support groups and patient groups. In Spain, there are 59 institutions or associations which provide support to families of children with ADHD. The management of 42 of those groups were contacted (it was not possible to contact the remaining 17) and asked to participate in the study. Nineteen agreed and contacted their member families to send them information about the study. 

A total of 138 informants participated in the study. Ninety-eight were parents of children aged between 6 and 12 years old with ADHD. The mean age of the fathers was 44.27 (*SD* = 6.48), and the mean age of the mothers was 42.59 (*SD* = 4.98). All of these parents’ children had been diagnosed with ADHD, with almost half of the diagnoses (43.9%) from clinical psychologists, 8.2% from educational psychologists, 3.1% from pediatricians, and 44.9% from neuro-pediatricians. 

A total of 40 children with ADHD participated in the study. They were aged between 7 and 12 years old (*M* = 9.68, *SD* = 1.38). Table 1 shows the information reported by the parents and children about gender and the characteristics of ADHD (type of presentation, severity, comorbidity, and pharmacological support).

### 2.2. Instruments

Data were collected using the scales from Bersabé et al. [25] and from the System for Evaluation of Children and Adolescents (Sistema de Evaluación de Niños y Adolescentes [SENA]) [26]. The scales from Bersabé et al. [25] were aimed at studying the relationships between parents and children. In the present study, we used both the parental version—in which parents respond to items about their own parenting styles—and the child version—in which the children report their perceptions of their parents’ parenting styles. Reponses are given on a five-point Likert scale (1—never, 2—not very often, 3—sometimes, 4—often, 5—always or almost always) based on how much the respondent feels that they (or their parents) perform the indicated behaviors.

The scale has two subscales, one for warmth–communication and criticism–rejection and another for rules and requirements. The rules and requirements subscale is made up of three factors: inductive discipline, rigid discipline, and indulgent discipline, referring to the ways that parents establish and demand conformance to rules. High scores in warmth–communication correspond to parents exhibiting higher levels of affection, warmth, interest, and communication with their children. High scores in criticism–rejection are associated with anger, lack of confidence, and lack of acceptance. In terms of the rules and requirements subscale, parents who inductively establish rules show more affection, interest, and communication with their children and criticize and reject them least. Rigid rule-setting is associated with parents expressing criticism, rejection, and lack of confidence in their children. Indulgent rule-setting indicates parents who encourage their children’s participation in creating rules, who do not use punishments, and who generally tolerate inappropriate behavior, finding it difficult to establish boundaries. The parental version has 48 items (10 items for warmth–communication, 10 items for criticism–rejection, 10 items for inductive rule-setting, 10 items for rigid rule-setting, and 8 items for indulgent rule-setting). The version for children has 96 items (20 for warmth–communication, 20 for criticism–rejection, 20 for inductive rule-setting, 20 for rigid rule-setting, and 16 for indulgent rule-setting).

In terms of reliability, the original version of the scale had adequate indices of internal consistency (Cronbach alpha coefficients between 0.60 and 0.90) [25]. In our sample, the values for Cronbach’s alpha were also adequate for the parent (warmth–communication α = 0.80, criticism–rejection α = 0.82, inductive α = 0.82, rigid α = 0.73, indulgent α = 0.65) and child versions (warmth–communication α = 0.89, criticism–rejection α = 0.55, inductive α = 0.90, rigid α = 0.82, indulgent α = 0.84).

The SENA was designed to detect a broad spectrum of emotional and behavioral difficulties [26]. There are various versions of the SENA, and in our study, we used the version for children aged 6 to 12 years old and the version for families. Responses are given on a five-point Likert-type scale, where the respondent indicates how often the described behavior appears (never or almost never, not often, sometimes, often, always or almost always). 

The SENA allows for the evaluation of a wide range of emotional and behavioral problems (depression, anxiety, hyperactivity, and impulsivity, defiant behavior, substance consumption, eating disorders, learning problems, etc.), contextual problems (problems with family, school, or peers), as well as assessing vulnerability (problems of emotional regulation, isolation, inflexibility, etc.), and psychological resources (self-esteem, social skills and integration, emotional intelligence). The variables considered for our study were attention, hyperactivity, anxiety, and emotional regulation. In the child version, the questionnaire is made up of 7 items for attention, 6 for hyperactivity, 6 for anxiety, and 6 for emotional regulation. In the parent version, there are 9 items for attention, 3 for hyperactivity, 11 for anxiety, and 7 for emotional regulation. High scores in SENA indicate the presence of symptomatology. High scores in attention suggest difficulties maintaining, regulating, and directing attention toward a task. High scores in hyperactivity–impulsivity are associated with excessive motor activity, usually accompanied by certain difficulties in inhibiting behavior and responding reflexively. High scores in anxiety indicate nervousness, intense restlessness, high physiological activation, and general subjective discomfort. Lastly, high scores in emotional regulation indicate difficulties controlling and managing emotions and mood.

The original version of the scale had satisfactory, or very satisfactory, coefficients of internal consistency (over 0.80 and 0.90) in line with the usual establishment of standards [27]. In our sample, the values for Cronbach’s alpha were also adequate for the parent (attention α = 0.81, hyperactivity α = 0.66, anxiety α = 0.90, emotional regulation α = 0.87) and child versions (attention α = 0.67, hyperactivity α = 0.90, anxiety α = 0.67, emotional regulation α = 0.89). 

### 2.3. Procedure

Data collection took place online, using two Google Forms (one for parents, the other for children). The process began on 21 March 2021 and ended on 2 November the same year. We contacted the management in 42 ADHD support and patient groups all over Spain to present the study objectives and through them made contact with the families of children with ADHD. These groups in Spain raise awareness and provide education, as well as supporting families of children with ADHD.

The groups were contacted via email initially and then via telephone. We held a meeting on the Microsoft Teams platform (1.7.00.3653) with members of the groups who had shown an interest. Ultimately, 19 groups agreed to participate in the study, and they were sent information to pass on to the families of children with ADHD, consisting of a link to a web page with the relevant information and the questionnaires for the parents and the children to complete.

The study was performed in compliance with the World Medical Association code of ethics (Declaration of Helsinki), reflecting the ethical principles for research with human beings [28] and was approved by the Ethical Committee of the Principality of Asturias (protocol code reference: CEISH-UPV/EHU, BOPV 32-2022). Before completing the survey, the participants were informed that their responses would be anonymous, and they were asked to agree for their results to be used in the study, ensuring informed consent from each subject. Parents gave consent at the same time for their children to participate. Informed consent was requested in the first section of the questionnaire, and the respondent could not continue answering the questions without it.

### 2.4. Statistical Analysis

The data were analyzed using SPSS 27. The results are presented separately for parents and children. In both cases, the descriptive and preliminary analyses are presented first, including a Pearson correlation matrix and the distribution of the variables. 

Subsequently, given the study objective, and in order to examine the influence of each variable associated with parenting styles on ADHD and emotional symptomatology, we performed linear regression analysis. The dependent variable was the symptomatology (attention, hyperactivity, anxiety, and regulation), while the independent variables were those associated with parenting styles (as reported by the parents in the first analysis and the children in the second): warmth–communication, criticism–rejection, inductive rule-setting, rigid rule-setting, and indulgent rule-setting.

## 3. Results

### 3.1. Descriptive Results 

Table 2 shows the families’ socio-demographic characteristics, including their socioeconomic levels, parental educational attainment, and occupational situation based on the information provided by parents.

### 3.2. Preliminary Results: Parents 

Table 3 shows the correlation matrix and distribution of the variables for the data collected from the parents. According to the values for asymmetry and kurtosis, the study variables met the criteria for normality, below 3 for asymmetry and below 10 for kurtosis [29].

The results of the correlation analysis indicated statistically significant correlations between some of the study variables. The parenting style variable Warmth–Communication correlated significantly and negatively with the variable Criticism–Rejection and positively with Inductive rule-setting. Criticism–Rejection in turn exhibited a significant negative correlation with Inductive rule-setting but positive correlations with Rigid and Indulgent rule-setting.

The Inductive rule-setting variable was significantly and negatively correlated with Rigid and Indulgent rule-setting, while Rigid rule-setting was significantly and positively correlated with Indulgent rule-setting.

Looking at the relationships between parenting styles variables and symptomatology, the variable Criticism–Rejection was significantly and positively related to Attention, Anxiety, and Emotional Regulation. Parents who perceived a higher level of criticism–rejection in their parenting also noted greater difficulties in attention, higher levels of anxiety, and worse emotional regulation. Looking at establishing rules, Rigid rule-setting was significantly and positively related to Emotional Regulation, highlighting that parents who were more rigid in setting rules noted worse levels of emotional regulation in their children.

Lastly, in terms of symptomatology, Attention was significantly and positively correlated with Hyperactivity, Anxiety, and Emotional Regulation. In addition, Hyperactivity was significantly and positively correlated with Emotional Regulation and Anxiety. Hence, the symptoms of attention, hyperactivity, anxiety and emotional regulation were very likely to be presented in combination.

### 3.3. Predictive Model for Symptomatology from Parents’ Perceptions of Parenting Style

Four regression models were specified, one for each dependent variable, to analyze the predictive power of the variables associated with parenting styles on Attention, Hyperactivity, Anxiety, and Emotional Regulation. All four models were statistically significant, reflecting that the parenting styles variables had predictive power over Attention, *F* (7, 89) = 2.13, *p* = 0.048; Hyperactivity, *F* (7, 89) = 2.85, *p* = 0.010; Anxiety, *F* (7, 89) = 2.20, *p* = 0.041; and Emotional Regulation, *F* (7, 89) = 3.94, *p* = 0.001. (see Table 4).

More specifically, the Criticism–Rejection variable exhibited predictive power on Attention, Anxiety, and Emotional Regulation (*p* = 0.004, *p* = 0.042, *p* < 0.001, respectively). However, although the model for Hyperactivity was statistically significant, the weight was in the age variable, which significantly and negatively predicted hyperactivity, indicating that the older the child, the lower the scores in the hyperactivity variable (*p* < 0.001).

As the *R*^2^ coefficient indicates, the variance explained was minimal for Attention and Anxiety and small for Hyperactivity (12%) and Emotional Regulation (18%).

### 3.4. Preliminary Results: Children

As Table 5 shows, looking at the information provided by the children, the values for asymmetry and kurtosis again complied with the criteria for normality. Examining the correlations, there was a positive, statistically significant correlation between the Warmth-Communication variable and Inductive rule-setting. There was also a significant negative correlation between Inductive rule-setting and Indulgent rule-setting.

Looking at parenting styles and symptomatology, the Warmth–Communication variable was statistically significantly and negatively correlated with Emotional Regulation, indicating that children who reported a parenting style based on warmth and communication also reported better emotional regulation. Rigid rule-setting was statistically significantly and positively related to the variables Hyperactivity, Anxiety, and Emotional Regulation.

In symptomatology, Attention was significantly and positively correlated with Hyperactivity, Anxiety, and Emotional Regulation. Hyperactivity was significantly and positively correlated with Anxiety and Emotional Regulation, and there was a significant positive correlation between Anxiety and Emotional Regulation.

### 3.5. Predictive Model for Symptomatology from Childrens’ Perceptions of Their Parents’ Parenting Styles 

We specified four regression models, one for each dependent variable. Three of the models were not statistically significant in predicting Attention, F (7, 32) = 1.841, *p* = 0.113; Hyperactivity, F (7, 32) = 1.874, *p* = 0.107; and Anxiety, F (7, 32) = 1.861, *p* = 0.109. However, the model for predicting Emotional Regulation was statistically significant, F (7, 32) = 2.448, *p* = 0.040, with children’s perceptions of parenting styles explaining 20.6% of the variance in their reported Emotional Regulation (see Table 6).

Considering each of the variables included in the model, Rigid rule-setting was statistically significant in predicting Hyperactivity (*p* = 0.049). In addition, Warmth–Communication (*p* = 0.028) and Rigid rule-setting (*p* = 0.041) were statistically significant in predicting Emotional Regulation.

## 4. Discussion

The aim of our study was to examine the extent to which variables associated with parenting styles as reported by parents of children with ADHD and by the children themselves would impact the presence of symptomatology associated with the disorder and other factors in the children’s emotional sphere.

The variables associated with the parenting styles that we considered in the study were warmth–communication, criticism–rejection, and inductive, rigid, or indulgent rule-setting. The goal was to determine how much predictive power these variables demonstrated for attention, hyperactivity, anxiety, and emotional regulation in children with ADHD.

To that end, we performed various regression analyses. The results indicated that parents’ perceptions of a parenting style based on criticism and rejection were a significant predictor of their perception of children’s attentional difficulties, anxiety, and emotional regulation issues. The four models analyzed were statistically significant, indicating that parenting style and the form of rule-setting had a small, though significant impact on the prediction of ADHD-associated symptomatology and emotional factors. The recent meta-analysis by Jendreizik et al. [15] showed that negative parenting practices (e.g., parental expression of displeasure and criticism) were consistently associated with more severe ADHD symptoms in children. However, in line with our results, they did not find positive parenting behaviors to be associated with symptom severity. According to Rogers et al. [30], parents of children with ADHD reported feeling less self-efficacy in their abilities to help their children and tended to use more coercive and punishment-based interactions regarding their children’s performance.

Another interesting piece of data from the regression model suggested that children’s age was a significant predictor of lower perceptions of hyperactivity on the part of parents. Along these lines, Biederman et al. [31] reported that age was significantly related to a reduction in ADHD symptomatology, with hyperactivity and impulsivity demonstrating the sharpest falls.

Consistent with this, children’s perceptions of rigid rule-setting significantly and positively predicted greater hyperactivity and difficulties in emotional regulation. In this case, only the emotional regulation prediction model was statistically significant, indicating that from the child’s perspective, the parenting style had more impact on their emotional stability than on attentional difficulties, hyperactivity, or anxiety. Meyer et al. [32] hypothesized that young children with inattentive or hyperactive symptoms may provoke a parenting style focused on control and protection. In their study with 102 parents of children with ADHD, they found that the relationship between child ADHD symptoms and child anxiety symptoms was mediated by parental overprotection (increased parental control, concerns regarding their child’s potential failure at tasks and new activities, and concerns that their child would elicit disapproval from others). Children may interpret this increased parental control as rigid rule-setting, which is associated with greater hyperactivity and emotional regulation problems in our study. 

It is worth noting that, unlike in the parents’ perceptions, children’s perceptions of a parenting style based on criticism and rejection demonstrated no association or predictive power for ADHD-associated symptomatology or emotional factors. Brinksma et al. [14] and Rothenberg [23] linked parental rejection as perceived by children with persistent internalizing and externalizing symptomatology in subsequent adolescence. Other longitudinal studies have found child ADHD symptoms to be predicted by earlier criticism/rejection, control, intrusiveness, warmth/sensitivity, inconsistent discipline, and involvement [20,33]. In terms of emotional factors, Mendo-Lázaro et al. [34] examined 1181 adolescents and found a clear relationship between practices based on criticism and rejection and emotional adjustment, from the children’s perceptions. 

Looking at the children’s perceptions, warmth–communication did demonstrate significant negative predictive power for emotional regulation difficulties, and in this case, warmth and communication may act as a protective factor against problematic levels of emotional regulation. Our results from both parent and child perspectives did not indicate that positive practices were associated with lower levels of ADHD symptoms. We have already noted that Jendreizik et al. [15] did not find positive parenting behaviors to be associated with the severity of child ADHD symptoms. In addition, Goodman et al. [19] reported that the association between positive and negative parenting practices and overall child functioning did not significantly differ. Looking at ADHD, Hawes et al. [20] reported that positive parenting (appropriate parental involvement) predicted lower levels of ADHD symptoms. In their meta-analysis, Jendreizik et al. [15] concluded that the greater importance of negative parenting behaviors than positive parenting behaviors might be specific to externalizing behavior problems in children. This conclusion gains traction in view of our results, which indicate that positive practices had an impact on emotional regulation but not on attention or hyperactivity.

### Limitations

It is important to note some of the limitations of the present study. Firstly, we did not consider parents’ or children’s different perceptions of fathers’ and mothers’ parenting styles. Although some studies have reported a narrowing of gender differences in parenting [34], it would be interesting to consider perceptions of fathers’ and mothers’ parenting styles and the consistency or inconsistency between the two. A second limitation relates to the type of measure used in the study. In line with previous research [14,23], we used parent-reported and child-reported information to measure parenting styles and children’s symptoms. However, children’s and parents’ perceptions may not be consistent with reality, or they may present a negative or positive pattern in their responses. Future studies should incorporate observational measures to assess parenting practices and children’s symptoms. Nevertheless, the most common measure in previous studies is parents’ and children’s ratings for assessing both parenting style and ADHD symptoms, and Rothenberg et al. [23] noted that major systematic reviews and meta-analyses have established that parent and child measures for the assessment of parental styles have demonstrated excellent reliability. Another limitation is related to the variables analyzed in the study. It is important to stress that the explanatory power of the variables in the regression model was low, with the highest being 20% (for the prediction of emotional regulation from children’s perceptions of parenting style), indicating that some other relevant variables might explain the variance. It would be useful for a study to analyze the profiles of interaction between children with ADHD and parents, as a person-centered approach might provide more depth when examining the results and how those profiles affect children’s attention, hyperactivity, anxiety, and emotional regulation. 

Finally, we did not consider the bidirectional influence of parent and child functioning. Some studies have highlighted the dynamic bidirectional influences between children and their environment/family. For example, Breaux and Harvey [33] found the presence of both parent and child effects in the relationship between child ADHD symptoms and family functioning. 

## 5. Conclusions 

Despite these limitations, we can conclude that parenting practices could explain, in part, symptomatology associated with ADHD and emotional factors. More specifically, as the literature has reported, and as we saw in our study, parenting practices based on criticism and rejection (as perceived by parents) have an effect on attentional difficulties, anxiety, and difficulties with emotional regulation (also as perceived by parents). However, children notice that practices based on warmth and communication benefit their emotional regulation. Furthermore, rigid rule-setting affects hyperactivity and emotional regulation in children’s eyes, although parents’ did not indicate an awareness that the way they set rules has an effect on their children’s symptoms. 

In this regard, one of the practical implications of the present study is that the impact of parental style as perceived by parents and children on ADHD symptomatology and other emotional factors differs in various aspects. While parents do not see a benefit from practices based on warmth–communication on their children’s emotional regulation, they do report a benefit (in terms of emotional regulation) of parental styles characterized by higher levels of affection, warmth, interest, and communication. At the same time, children see that rigid formulas for relationships in the family, placing great importance on obedience and the use of punishment, negatively affect their hyperactivity and again their emotional regulation. Furthermore, unlike their parents, the children did not report that criticism and rejection affected their attention, anxiety, or emotional regulation, and they gave more weight to the positive effect of warmth and communication than to the negative effect of criticism–rejection. Parents should consider that, for their children, the most important aspect is a warm, supportive relationship and attention to their needs, which will have a positive impact on their emotional regulation and development. 

An additional practical implication, given that these variables associated with parenting styles (such as criticism–rejection) are so important in the progression of ADHD, is that programs should include training for parents on parenting practices based on warmth, affection, communication, and dialog. As Jendreizik et al. [15] indicated, commonly used interventions with children with ADHD should include parent-centered interventions to address family dynamics, practices, and behaviors. 

We must not ignore the fact that ADHD has an important hereditary component and that parents of children with ADHD may also be suffering from ADHD symptoms. Park et al. [35] emphasized that parental ADHD symptoms are associated with more negative parenting behaviors. When ADHD is detected in a child, we should not restrict ourselves to only working with the child; professionals have to provide specific interventions for parents. In terms of the etiology of the disorder, an interaction between polygenic and environmental risk factors is assumed to play an important role that we might be able to minimize by dealing with risk factors, such as family interactions, with interventions that consider the developmental context. 

In sum, our study attempted to use parents’ and children’s perspectives to provide a better understanding of which parental practices are associated not only with ADHD but also with emotional factors. We aimed to contribute to the study of the different aspects that affect child development and establish the importance of intervention approaches that consider family dynamics. 

## Figures and Tables

**Table 1:** Characteristics of the sample.

	Informant			
	Parents (*N* = 98)		Children (*N* = 40)	
	*n*	%	*n*	%
Gender				
Girls	29	29.6	14	35
Boys	69	70.4	26	65
ADHD—Presentation				
Combined	45	45.9	21	52.5
Inattentive	27	27.6	8	20
Not Determined	26	26.5	11	27.5
ADHD—Severity				
Mild	26	26.5	9	22.5
Moderate	44	44.9	24	60
Severe	28	28.6	7	17.5
Comorbidity				
Learning Disorder	19	19.4	7	17.5
Behavior Disorder	8	8.2	4	10
Physical Disorder	10	10.2	5	12.5
Pharmacological Support				
Yes	75	76.5	30	75
No	23	23.5	10	25

**Table 2:** Sociodemographic characteristics of the sample.

	*n*	%
Socio-economic level		
Low	13	13.3
Medium	82	83.7
High	2	2
Fathers’ educational attainment		
No qualifications	8	8.2
Basic	27	27.6
Post-compulsory	46	46.9
Graduate	14	14.3
Postgraduate	3	3.1
Mothers’ educational attainment		
No qualifications	0	0
Basic	11	11.2
Post-compulsory	45	45.9
Graduate	32	32.7
Postgraduate	10	10.2
Occupational situation		
Inactive	2	2
One parent working	26	26.5
Both parents working	70	71.2
Family		
Single-parent	5	5.1
Two-parent family	89	90.8
Blended family	4	4.1

**Table 3:** Pearson correlation matrix and distribution of the variables collected from parents’ information.

	1	2	3	4	5	6	7	8	9
1. Warmth– Communication	–								
2. Criticism–Rejection	−0.29 **	–							
3. Inductive	0.50 ***	−0.28 **	–						
4. Rigid	−0.14	0.41 ***	−0.22 *	–					
5. Indulgent	−0.06	0.21 *	−0.20 *	0.22 *	–				
6. Attention	−0.15	0.30 **	−0.06	0.07	0.04	–			
7. Hyperactivity	−0.00	−0.04	0.07	−0.01	−0.09	0.46 ***	–		
8. Anxiety	−0.04	0.27 **	−0.13	0.09	0.18	0.31 **	0.19	–	
9. Regulation	0.00	0.41 ***	−0.06	0.27 **	0.15	0.47 ***	0.33 **	0.49 ***	─
*M*	4.55	2.04	4.33	2.73	1.73	3.90	3.82	3.14	3.47
*SD*	0.40	0.54	0.46	0.62	0.45	0.64	0.94	0.96	0.98
Asymmetry	−1.11	0.75	−0.70	0.06	0.83	−0.59	−0.63	−0.00	−0.41
Kurtosis	2.05	0.85	0.16	−0.11	1.54	0.55	−0.02	−0.67	−0.64
Minimum	2.90	1.10	2.80	1.30	1	1.78	1	1	1.14
Maximum	5	4	5	4.30	3.38	5	5	5	5

Note. *** *p* < 0.001, ** *p* < 0.01, * *p* < 0.05.

**Table 4:** Regression models for predicting Attention, Hyperactivity, Anxiety, and Regulation from parents’ perceptions of parenting styles.

	Attention		Hyperactivity		Anxiety		Emotional Regulation	
	β	*t*	β	*t*	β	*t*	β	*t*
1. Sex	−0.28	−1.86	−0.19	−0.87	−0.31	−1.38	−0.33	−1.56
2. Age	−0.07	−1.50	−0.27	−4.32 ***	0.08	1.17	−0.09	−1.39
3. Warmth– Communication	−0.08	−0.39	−0.09	−0.35	0.24	0.86	0.39	1.41
4. Criticism–Rejection	0.40	2.94 **	0.09	0.45	0.42	2.07 *	0.76	3.87 ***
5. Inductive	0.13	0.77	0.29	1.23	−0.21	−0.86	0.09	0.39
6. Rigid	−0.03	−0.25	0.06	0.39	−0.04	−0.24	0.21	1.32
7. Indulgent	−0.06	−0.41	−0.23	−1.08	0.28	1.24	0.08	0.36
*R* ^2^	0.08		0.12		0.08		0.18	

Note. *t* = Student *t*-test; *R*^2^ = variance explained. *** *p* < 0.001, ** *p* < 0.01, * *p* < 0.05.

**Table 5:** Pearson correlation matrix and distribution of the variables collected from children’s information.

	1	2	3	4	5	6	7	8	9
1. Warmth– communication	–								
2. Criticism–rejection	−0.19	–							
3. Inductive	0.72 ***	0.01	–						
4. Rigid	−0.02	0.26	−0.01	–					
5. Indulgent	−0.08	−0.05	−0.33 *	0.25	–				
6. Attention	−0.05	0.16	−0.21	0.24	0.24	–			
7. Hyperactivity	−0.07	0.22	−0.10	0.43 **	0.24	0.67 ***	–		
8. Anxiety	−0.31	0.29	−0.27	0.36 *	0.28	0.46 **	0.63 ***	–	
9. Regulation	−0.32 *	0.18	−0.17	0.41 **	0.25	0.51 ***	0.76 ***	0.69 ***	─
*M*	4.22	2.75	4.16	2.88	1.91	3.36	3	2.87	2.92
*SD*	0.54	0.36	0.64	0.66	0.58	1.27	1.11	1.09	1.02
Asymmetry	−0.43	0.48	−0.47	−0.22	0.63	0.93	−0.13	0.89	0.15
Kurtosis	−0.97	−0.81	−0.74	−0.16	0.57	4.27	−0.81	0.74	−0.85
Minimum	3.15	2.20	2.60	1.45	1	1	1	1	1
Maximum	4.95	3.55	5	4.30	3.63	8.14	5	6.17	5

Note. *** *p* < 0.001, ** *p* < 0.01, * *p* < 0.05.

**Table 6:** Regression models for prediction of Attention, Hyperactivity, Anxiety, and Regulation from parents’ perceptions of parenting styles.

	Attention		Hyperactivity		Anxiety		Emotional Regulation	
	β	*t*	β	*t*	β	*t*	β	*t*
1. Sex	0.65	1.62	0.21	0.59	0.04	0.12	−0.11	−0.35
2. Age	−0.27	−1.90	−0.20	−1.59	−0.04	−0.31	−0.13	−1.22
3. Warmth– Communication	0.57	1.05	0.04	0.09	−0.41	−0.87	−0.97	−2.30 *
4. Criticism–Rejection	0.56	0.99	0.34	0.68	0.57	1.16	−0.08	−0.19
5. Inductive	−0.89	−1.87	−0.25	−0.60	−0.12	−0.28	0.38	1.02
6. Rigid	0.21	0.70	0.56	2.04 *	0.42	1.55	0.51	2.12 *
7. Indulgent	0.24	0.67	0.21	0.67	0.36	1.12	0.32	1.15
*R* ^2^	0.131		0.136		0.134		0.206	

Note. *t* = Student *t*-test; *R*^2^ = variance explained. * *p* < 0.05.

## Data Availability

The data presented in this study are available on request from the corresponding author. The data are not publicly available due to privacy and ethical reasons.
